# Targeting cellular mitophagy as a strategy for human cancers

**DOI:** 10.3389/fcell.2024.1431968

**Published:** 2024-07-05

**Authors:** Yuming Dong, Xue Zhang

**Affiliations:** ^1^ School of Stomatology, China Medical University, Shenyang, China; ^2^ The VIP Department, School and Hospital of Stomatology, China Medical University, Shenyang, China

**Keywords:** mitophagy, cancer, mitochondria, PINK1, stemness

## Abstract

Mitophagy is the cellular process to selectively eliminate dysfunctional mitochondria, governing the number and quality of mitochondria. Dysregulation of mitophagy may lead to the accumulation of damaged mitochondria, which plays an important role in the initiation and development of tumors. Mitophagy includes ubiquitin-dependent pathways mediated by PINK1/Parkin and non-ubiquitin dependent pathways mediated by mitochondrial autophagic receptors including NIX, BNIP3, and FUNDC1. Cellular mitophagy widely participates in multiple cellular process including metabolic reprogramming, anti-tumor immunity, ferroptosis, as well as the interaction between tumor cells and tumor-microenvironment. And cellular mitophagy also regulates tumor proliferation and metastasis, stemness, chemoresistance, resistance to targeted therapy and radiotherapy. In this review, we summarized the underlying molecular mechanisms of mitophagy and discussed the complex role of mitophagy in diverse contexts of tumors, indicating it as a promising target in the mitophagy-related anti-tumor therapy.

## Introduction

**FIGURE 1:**
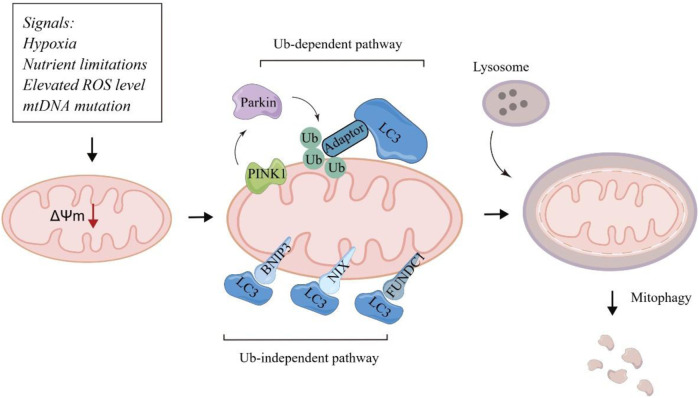
Major molecular mechanisms of mitophagy.

Mitochondria are highly complex and dynamic organelles that regulate cellular metabolism in biosynthesis, bioenergetics, redox homeostasis and signaling functions (Zong et al., 2016). Notably, mitochondrial biogenesis is commonly upregulated in tumors, and mitochondria widely participates in different stages of tumorigenesis (Wallace, 2012). The maintenance of mitochondrial integrity and functional network is critical for tumors to survive and adapt to the hypoxic and nutrient-limited tumor microenvironment. Mitophagy is a specific type of autophagy to eliminate damaged and dysfunctional mitochondria, determining the number and quality of mitochondria (Lu et al., 2023). Under hypoxic and stressful conditions, mitochondria will undergo depolarization to remove damaged mitochondria through autophagic mechanism, which plays a key role in regulating the malignant biological behaviors of tumor cells (Ferro et al., 2020; Panigrahi et al., 2020). Typical mitophagy pathways include ubiquitin-dependent pathways mediated by PTEN induced kinase 1 (PINK1) -Parkin and non-ubiquitin dependent pathways mediated by mitochondrial autophagic receptors such as NIX, BNIP3, and FUNDC1 (Wang et al., 2023a) (Figure 1). In this review, we discussed the underlying molecular mechanisms of mitophagy and highlighted the complex role of mitophagy in diverse contexts of tumors, indicating it as a promising target in the mitophagy-related anti-tumor therapy (Table 1).

**TABLE 1:** Summarizing mitophagy-related anti-tumor therapy.

Drug	Effect	Combined therapy	Cancer types	References
Mdivi-1	Inhibiting the myoferlin-related ROS production and restoring cell growth	Iron chelators	Pancreatic ductal adenocarcinoma	Rademaker et al. (2022)
Oroxylin A	Overcoming drug resistance by downregulating PINK1/Parkin-mediated mitophagy	–	Hepatocellular carcinoma	Yao et al. (2022)
STM2457	Targeting METTL3 to overcome drug resistance by downregulating PINK1/Parkin-mediated mitophagy	–	Small cell lung cancer	Sun et al. (2023)
Chloroquine	Eradicating persister cells and promoting complete response to MAPK inhibitors	MAPK inhibitors	Lung adenocarcinoma	Li et al. (2023)
Hydroxychloroquine	Inhibiting PINK1/Parkin-mediated mitophagy, tumor growth and metastasis	Lenvatinib	Hepatocellular carcinoma	

### Mitophagy: elimination of target mitochondria through selective autophagy

It has been well-established that the recognition of targeted mitochondria by the autophagosome occurs mainly through LC3 adapters in an ubiquitin-dependent pathways mediated by PINK1/Parkin or non-ubiquitin dependent pathways mediated by mitochondrial autophagic receptors.

### Ubiquitin-dependent pathways mediated by PINK1/Parkin

PINK1 is a serine/threonine kinase that functions as a sensor of mitochondrial damage to cooperate with Parkin, a cytosolic E3 ubiquitin ligase, to induce mitophagy by targeting damaged mitochondria for the lysosomal degradation (Han et al., 2023). When mitochondria are undamaged, PINK1 is imported to the inner mitochondrial membrane (IMM) via the complex of translocase complexes. Once located at the IMM, PINK1 is cleaved for degradation. When mitochondria are impaired (indicated by the accumulation of unfolded mitochondrial proteins or altered mitochondrial membrane potential), the full-length PINK1 is accumulated and stabilized by impairing import to the IMM (Nguyen et al., 2016). PRKN is further phosphorylated and activated by PINK1 at Ser65 (Kane et al., 2014). Upon being phosphorylated, PRKN further regulates diverse proteins with K11- and K63-linked UB chains to recruit autophagy receptors and remove damaged mitochondria to mediate mitophagy. PINK1 was strongly correlated with a poor prognosis in cancer patients (Zheng et al., 2023a).

### Non-ubiquitin dependent pathways mediated by mitochondrial autophagic receptors

BNIP3 and its homolog BNIP3L/NIX are outer mitochondrial membrane (OMM) proteins and function as mitophagy receptors that mediates mitophagy under stresses, particularly hypoxia. Under hypoxia, BNIP3 and BNIP3L are activated and localized to the OMM via the carboxy-terminal transmembrane domain. The transmembrane domain with a glycine zipper that is essential for the homo-dimerization of BNIP3, which is important for its interplay with LC3 for mitophagy (Zhang and Ney, 2009). Targeting the BNIP3-mediated mitophagy has been found to be combined with anti-CD30 antibody to improve the prognosis of CD30^+^ EBV + diffuse large B-cell lymphoma patients (Wang et al., 2024). Collectively, BNIP3 has emerged as a promising therapeutic and diagnostic target in multiple cancers. Like BNIP3 and BNIP3L/NIX, FUNDC1 promotes hypoxia-induced mitophagy. FUNDC1 integrates into the OMM and its LC3 interaction regions motif could project into the cytosol to interact with LC3 (Poole and Macleod, 2021). Targeting these mitochondrial autophagic receptors may provide novel and promising anti-tumor strategy.

### Functional network of mitophagy and mitochondrial dynamics

Mitochondria are highly dynamic organelles that constantly undergo fusion and fission. Mitochondrial fusion integrates two mitochondria at the outer and inner membrane interfaces primarily via Mitofusin 1 (MFN1), Mitofusin 2 (MFN2), and Optic atrophy protein 1 (OPA1). Mitochondrial fission is the process whereby a mitochondrion divides into two mitochondria, which is mediated by Dynamin-related protein 1 (DRP1). Mitochondrial fusion and fission have been found to be critical for removing damaged mitochondria by mitophagy. MFN2 overexpression induces mitochondrial fusion and leads to increased mitophagy in pancreatic adenocarcinoma cells (Yu et al., 2019). Jiang et al. found that blocking mitochondrial recruitment of MFN2 reduces formation of the PINK1/MFNA2/Parkin complex required for initiation of mitophagy (Jiang et al., 2022a). Herein, mitochondrial fusion and fission play a vital role in cellular mitophagy.

### Role of cellular mitophagy in human cancers

**FIGURE 2:**
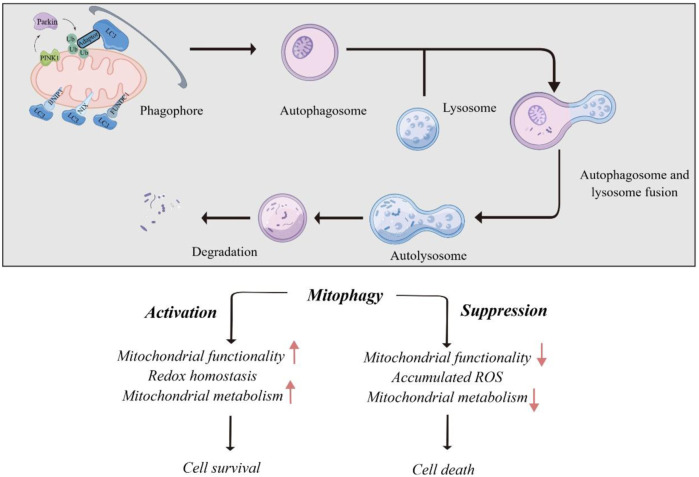
Role of mitophagy in human cancers.

Mitophagy plays a role in both cell death and survival. Excessive mitophagy leads to the loss of functional mitochondria, leading to insufficient energy supply and cell death. On the other hand, mitophagy promotes cell survival by eliminating damaged mitochondria to adapt to the environment. In malignant tumors, mitophagy is involved in the abnormal activation and proliferation of cancer cells, suggesting that it can have both tumorigenic and tumor-suppressive effects (Chang et al., 2017). The balance between these two effects coordinates to determine tumor development or apoptosis (illustrated in Figure 2). From this perspective, a novel anti-tumor therapy can not only inhibit the mitophagy of cancer cells for anti-tumor effect, but also enhance the mitophagy of normal cells and remove damaged mitochondria, to maintain the stability and function of mitochondrial genome. Therefore, further understanding of the molecular mechanism of mitophagy signaling pathway is expected to provide new ideas for the formulation of clinical anti-tumor therapeutic strategies.

### Role of mitophagy in tumor proliferation and metastasis

In some cancers, mitophagy was found to inhibit tumor development. G protein-coupled receptor 176 (GPR176) regulates mitophagy via the cAMP/PKA/BNIP3L axis, leading to initiation and progression of colorectal cancer. Mechanistically, the recruitment of the G protein GNAS intracellularly is essential for the transduction of GPR176-mediated signals (Tang et al., 2023). In gastric cancer, gamma-glutamyltransferase 7 (GGT7) is a tumor-suppressive regulator by interacting with RAB7 and re-locating RAB7 to cytoplasm, leading to enhanced mitophagy and reduced ROS production (Wang et al., 2022). Unc-51 like kinase 1 (ULK1) deficiency has been found to enhance invasive potentials and osteolytic bone metastasis of breast tumors via attenuating mitophagy. Mechanistically, ULK1 inhibition suppresses mitophagy under hypoxia, leading to accumulated damaged mitochondria and NLRP3 inflammasome activation, which ultimately alters cytokine secretion for osteoclast differentiation and bone metastasis (Deng et al., 2021).

On the contrary, multiple studies proposed that enhanced mitophagy promotes tumor proliferation and metastasis. BCL2 like 13 (BCL2L13) targeted DNM1L at the Ser616 site to promote mitochondrial fission and mitophagy, which ultimately enhance the proliferation and invasion of glioblastoma cells (Wang et al., 2023b). In triple-negative breast cancer, divalent metal transporter 1 (DMT1) induces mitochondrial iron translocation via endosome-mitochondria interactions. DMT1 knockdown elevates labile iron pool levels and activates PINK1/Parkin-dependent mitophagy to promote the outgrowth of lung metastatic nodules. These findings reveal a DMT1-dependent pathway connecting endosome-mitochondria interactions to mitochondrial iron translocation and metastatic fitness of breast cancer cells (Barra et al., 2024).

### Role of mitophagy in tumor stemness

Mitochondria plays a key role in the stemness maintenance and differentiation of cancer stem cells (CSCs) (Zheng et al., 2023b). It has been proposed that mitophagy is highly active in CSCs. Deregulation of ADAR1 is closely correlated with self-renewal of liver CSCs (Jiang et al., 2022b). Enhanced mitophagy has been observed in ADAR1-enriched liver CSCs. In addition, GLI1 editing promotes a metabolic shift to oxidative phosphorylation to sustain stemness through PINK1/Parkin-mediated mitophagy in hepatocellular carcinoma (HCC), therefore enhancing metastatic potential and sorafenib resistance of HCC. The highly active mitophagy has also been identified as a key feature of lung CSCs, driving metabolic reprogramming via the Notch1/AMPK axis to induce lung CSC expansion (Liu et al., 2023a). Hyperactivated mitophagy in lung CSCs leads to increased mitochondrial DNA (mtDNA) content in the lysosome. The mtDNA in lysosomal fractions from CSCs was highly oxidized and significantly higher than that from non-CSC cells. Lysosomal mtDNA further serves as an endogenous ligand for Toll-like receptor 9 (TLR9) to enhance the interaction between Notch1 and AMPK to promote lysosomal AMPK activation. Lysosomal mtDNA-dependent TLR9 signaling induces Notch1/AMPK activation to promote mitochondrial metabolism in CSCs. Targeting the TLR9-Notch-AMPK pathway in high-mitophagy lung tumors reduces the CSC pool and blocks tumor growth for non-small cell lung cancer treated with chemotherapy. (Liu et al., 2023b). In glioblastoma stem cells (GSCs), platelet-derived growth factor (PDGF) promotes m6A accumulation to regulate mitophagy. PDGF ligands induce EGR1 transcription to upregulate methyltransferase-like 3 (METTL3) to sustain self-renewal of GSC. Targeting the PDGF/METTL3 axis could impair GSC mitophagy in an OPTN-dependent manner (Lv et al., 2022). Clusterin (CLU) has been found to exert its mitophagy-specific role in oral CSCs. CLU can regulate mitochondrial fission by activating the serine/threonine kinase AKT, triggering the phosphorylation of Drp1 at serine 616 residue and thus initiating mitochondrial fission. CLU-induced mitophagy enhances self-renewal capability of oral CSCs through mitophagic degradation of MSH homeobox 2 and prevents its nuclear translocation to inhibit SOX2 activity (Praharaj et al., 2023). Interferon-stimulated gene 15 (ISG15) and protein ISGylation is upregulated in pancreatic CSCs for maintaining their metabolic plasticity (Alcalá et al., 2020). ISG15 abrogation inhibits ISGylation, oxidative phosphorylation and mitophagy to impair self-renewal and tumorigenic ability of pancreatic CSCs. Thus, ISGylation is critical for mitophagy to clean dysfunctional mitochondria and maintain pancreatic CSCs (Alcalá et al., 2020).

### Role of mitophagy in tumor chemoresistance

Mitophagy plays a multifaceted role in tumor chemoresistance. Multiple studies have elucidated that enhanced mitophagy promote chemoresistance in specific cancer types. In small cell lung cancer (SCLC), METTL3 confers SCLC cells resistance to chemotherapy by upregulating mitophagy. METTL3 induces m6A methylation of DCP2 to induce PINK1/Parkin-mediated mitophagy and promote chemotherapy resistance. METTL3 inhibitor STM2457 could reverse the chemoresistance of SCLC (Sun et al., 2023). Stomatin-like protein 2 (STOML2) is located in the IMM and is highly expressed in cancer cells. STOML2 stabilizes PARL and prevents gemcitabine-mediated PINK1-dependent mitophagy to reduce the chemoresistance of pancreatic cancer, making STOML2-targeted therapy as a potential strategy for gemcitabine sensitization (Qin et al., 2023). In contrast, various studies illustrated mitophagy may inhibit chemoresistance in some cancers. CRL4CUL4A/DDB1, a well-defined E3 ubiquitin ligase, was significantly upregulated in cisplatin-resistant ovarian cancer cells by inhibiting mitophagy. Downregulation of CRL4CUL4A/DDB1, promotes mitophagy by regulating the PINK1/Parkin axis, DRP1 dephosphorylation at Ser637, and the interplay between DRP1 and voltage-dependent anion channel 1 (VDAC1), ultimately driving mitochondrial fission and mitophagy in chemotherapy-resistant ovarian tumor cells (Meng et al., 2022).

### Role of mitophagy in resistance to targeted therapy

Drug-tolerant persister (DTP) tumor cells leads to tumor relapse (Dhanyamraju et al., 2022). The efficacy of EGFR-TKIs is limited due to drug resistance. In combination of circular RNA IGF1R (cIGF1R) with EGFR-TKIs could synergize to block tumor re-growth after drug withdrawal. The cIGF1R encodes a peptide that reduces Parkin-induced ubiquitination of VDAC1 to block mitophagy, indicating a molecular switch that transiting of DTP to apoptosis (Wang et al., 2023c). BH3 mimetic antagonists to BCL-2 and MCL-1 has been considered as an anti-tumor strategy to induce cell death in acute myeloid leukemia (AML), and resistance to BH3 mimetics has been identified as a critical clinical problem (Bhatt et al., 2020). AML cells resistant to BH3 mimetics is correlated with high influx of mitophagy, and pharmacologic inhibition of autophagy could sensitize AML cells to BH3 mimetics. MFN2 has been identified as a regulator of mitophagy and functions as a receptor for Parkin onto the damaged mitochondria, which leads to resistance to BH3 mimetics in AML (Glytsou et al., 2023). Targeting MFN2 could synergize with BH3 mimetics by blocking mitophagy and inducing apoptosis in AML. Lenvatinib is a standard therapy option for advanced HCC. In HCC, LINC01607 induces protective mitophagy by upregulating P62, which reduces ROS levels and induces drug resistance. LINC01607 knockdown in combination with lenvatinib could reverse resistance *in vivo* (Zhang et al., 2023).

### Role of mitophagy in resistance to radiotherapy

Enhanced DNA damage repair is essential for radiation resistance in tumor cells, and mitophagy functions as a critical upstream signal to enhance radiation-mediated DNA damage by regulating mitophagy proteins. SIRT3 was found to be upregulated in colorectal tumor cells and leads to PINK1/Parkin-mediated mitophagy. Hyperactivated mitophagy promotes DNA damage repair, therefore inducing radiation resistance. Mechanistically, mitophagy leads to RING1b downregulation and impaired ubiquitination of histone H2A to enhance DNA damage repair (Wei et al., 2023). In melanoma, hyperactivated mitophagy in combination of radiation could augment DNA damage and inhibit tumor progression (Ren et al., 2023). Mitophagy is also essential for ferroptosis under radiation. Radiation leads to the degradation of the peri-droplet mitochondria by lysosomes to release free fatty acids and increase lipid peroxidation for ferroptosis (Yang et al., 2023).

### Role of mitophagy in anti-tumor immunity

Mitophagy plays a key role in the maintenance of mitochondrial function, ensuring the effective participation of specific immune cells and the realization of cell-specific immunomodulatory functions. In addition, mitophagy can further regulate immune function by inhibiting the production of mitochondrial components that regulate immune response (Song et al., 2020).

In the process of immune response, T cells highly depend on mitochondria to support their evolving metabolic requirements. Maintenance of mitochondrial health requires removal of damaged mitochondria through mitophagy via PINK1/Parkin- or BNIP3L/NIX-mediated pathways. Franco et al. explored the function of mitochondrial quality control in memory T cell responses and found that mitophagy machinery orchestrates survival and metabolic dynamics required for memory T cell formation (Franco et al., 2023). Urolithin A, generated from gut microbiome from foods, has been found to improve mitochondrial health. Urolithin A can enhance the anti-tumor CD8^+^ T cell immunity *in vivo*. It has been found that urolithin A-induced T memory stem cell formation depends on PINK1-mediated mitophagy to induce release of PGAM5 into the cytoplasm. Cytosolic PGAM5 dephosphorylates β-catenin to activate Wnt pathway and mitochondrial biogenesis (Denk et al., 2022). Gupta et al. found that NIX-mediated mitophagy is essential for effector memory formation in T cells. Deficiency in NIX-dependent mitophagy results in HIF1α accumulation and metabolic alteration to impair ATP production during effector memory formation in T cells (Gupta et al., 2019).

Therapeutic response to immunochemotherapy is closely correlated with subcellular re-distribution of PD-L1. Recent study has elucidated that the distribution pattern of PD-L1 is determined by ATAD3A/PINK1-mediated mitophagy. PINK1 could recruit PD-L1 to mitochondria for degradation, while paclitaxel upregulates ATAD3A to impair proteostasis of PD-L1 by blocking PINK1-mediated mitophagy. ATAD3A/PINK1-mediated mitophagy determines the efficacy of immunochemotherapy by PD-L1 re-localization, which is a promising target for promoting the therapeutic responses to immunochemotherapy (Xie et al., 2023).

### Role of mitophagy in metabolic reprogramming

Cellular mitophagy is essential for maintaining functional mitochondria, which is a prerequisite of tumor cells to mediate metabolic shift from glycolysis towards oxidative phosphorylation. In lung adenocarcinoma cells and organoids, PINK1 has been found to be upregulated to sustain mitochondrial homeostasis during DTP generation, and PINK1-induced mitophagy drives DTP production upon MAPK inhibition. PINK1-induced mitophagy promotes DTP cell survival, while MAPK inhibition leads to MYC-regulated upregulation of PINK1, therefore activating mitophagy of DTP cells. Mitophagy inhibition via chloroquine or PINK1 abrogation could enhance the therapeutic response to MAPK inhibitors (Li et al., 2023). Targeting iron metabolism in tumor cells is an emerging opportunity for anti-tumor therapeutics, and iron is an essential component involved in the electron transport chain within mitochondria. Sandoval-Acuña et al. found that targeting mitochondrial iron metabolism by deferoxamine inhibits tumor progression by inducing mitochondrial dysfunction and mitophagy (Sandoval-Acuña et al., 2021).

### Role of mitophagy in cancer-associated fibroblasts

Among the stromal cells in the tumor microenvironment, cancer-associated fibroblasts (CAFs) are the most abundant and actively involved in tumor progression through versatile interplay with other cell types in the TME. Blocking mitophagy by targeting Parkin in the CAFs impairs tumor growth *in vivo*. Autophagy deficiency in CAFs also enhances proline biosynthesis through mitophagy-induced regulation of NAD kinase 2 (Bai et al., 2023). TNBC cells with integrin beta 4 (ITGB4) overexpression provided with ITGB4 protein via exosomes to induce BNIP3L-mediated mitophagy in CAFs. Co-culture experiment revealed that the ITGB4-meidated mitophagy is impaired in CAFs by ITGB4 inhibition in MDA-MB-231 cells, and ITGB4-positive CAF-conditioned medium could promote malignant behaviors of TNBC cells (Sung et al., 2020). Thus, targeting mitophagy of CAFs may be a promising strategy for CAF-targeted anti-tumor intervention.

### Role of mitophagy in hypoxic tumor microenvironment

Hypoxic microenvironment is a common feature of solid tumors, and hypoxia exerts great effect on the malignant behavior of tumor cells (Chen et al., 2023). Upon hypoxia, LYPLA1-mediated depalmitoylation of glycerophosphocholine phosphodiesterase 1 (GPCPD1) induces GPCPD1 translocating from cytoplasm to mitochondria. Notably, mitochondrial GPCPD1 binds to and interacts with VDAC1 to impair the oligomerization of VDAC1. VDAC1 monomer further recruits Parkin-induced poly-ubiquitination to induce mitophagy and promote progression of TNBC (Liu et al., 2023a). Under the hypoxic condition, mitophagy receptor FUNDC1 accumulates at the mitochondria-associated membranes to stabilize the FUNDC1/ULK1 complex for cell survival and tumor development (Ponneri Babuharisankar et al., 2023).

### Role of mitophagy in ferroptosis

Ferroptosis is an iron-dependent type of programmed cell death closely correlated with lipid peroxidation (Jiang et al., 2021). Recent study revealed that the inter between mitochondrial integrity and ferroptosis determines the cell survival. Myoferlin is an oncoprotein that upregulated in pancreatic ductal adenocarcinoma and participates in the regulation of cell membrane biology. Pharmacological inhibition of myoferlin via WJ460 could induce mitophagy and ROS accumulation culminating with lipid peroxidation and apoptosis-independent cell death. WJ460 caused a reduction of the abundance of ferroptosis core regulators xc-cystine/glutamate transporter and GPX-4. Mitophagy inhibitor Mdivi1 and iron chelators inhibited the myoferlin-related ROS production and restored cell growth. A synergic effect between ferroptosis inducers, erastin and RSL3, and WJ460 (Rademaker et al., 2022). Oroxylin A (OA), a novel CDK9 inhibitor, showed strong therapeutic potential against HCC and a striking capacity to overcome drug resistance by downregulating PINK1-PRKN-mediated mitophagy. CDK9 inhibitors promoted dephosphorylation of SIRT1 and promoted FOXO3 protein degradation, which was regulated by its acetylation, leading to the transcriptional repression of FOXO3-driven BNIP3 and impairing the BNIP3-mediated stability of the PINK1 protein (Yao et al., 2022).

### Future directions for mitophagy-based anti-tumor strategy

One challenge in the mitophagy-based drug development is specificity. The concept of specificity is required for optimizing drug efficacy and reducing adverse events. However, current mitophagy-based drugs are non-selective and do not meet this criterion. Chloroquine and hydroxychloroquine are basic amphiphiles that function in the lysosome and impair lysosomal function as the main mechanism of action. High-dose hydroxychloroquine given for cancer therapy induces irreversible side effect of retinal toxicity (Leung et al., 2015). Chloroquine and hydroxychloroquine have similar properties of pharmacokinetic with high volume of distribution and prolonged plasma half-lives (Schrezenmeier and Dörner, 2020). It has been proposed that the reformulation of chloroquine and hydroxychloroquine is required to benefit their pharmacokinetic and safety to support the use of chloroquine and hydroxychloroquine for the treatment of cancer.

Nanoparticle administration is useful for overcoming poor pharmacokinetic and toxicity as well as promoting site-specific administration by improving the solubility of hydrophobic drugs, preventing drugs from degradation, and altering tissue distribution (Amreddy et al., 2018). In the treatment of cancer, a variety of nanomedicines have been developed with clinical approval, which greatly enhance the safety and effectiveness of anti-tumor drugs. Therefore, nanoparitcle reformulation of mitophagy-based drugs, such as chloroquine and hydroxychloroquine, increase exposure of target tissues relative to off-target tissues and reduce off-target toxicity (Stevens et al., 2020).

## Conclusion

It has been well-established that the regulation of mitophagy may be a new direction for the treatment of tumors. Through in-depth analysis of the potential molecular mechanism of mitophagy, it may provide theoretical basis for further research on novel anti-tumor therapy. The importance of mitophagy in tumorigenesis and development has been well established. Mitophagy plays a key role in regulating intracellular environmental homeostasis and clearing damaged mitochondria, thereby regulating mitochondrial function and oxidative stress. Therefore, certain mitophagy inhibitors or activators may have great potential for anti-tumor strategy. Therefore, it is of great significance to explore the influence of mitophagy on tumorigenesis and development. In future studies, through proteomics, transcriptomics, metabolomics and sing-cell sequencing technologies to further explore the molecular mechanisms regulating mitophagy, it will help to explore potential pharmacological small molecules targeting mitochondrial autophagy, so as to provide more effective anti-tumor treatment.
